# Rapid Quantum Magnetic IL-6 Point-of-Care Assay in Patients Hospitalized with COVID-19

**DOI:** 10.3390/diagnostics12051164

**Published:** 2022-05-07

**Authors:** Johnny Atallah, Dakota Archambault, Jeffrey D. Randall, Adam Shepro, Lauren E. Styskal, David R. Glenn, Colin B. Connolly, Katelin Katsis, Kathleen Gallagher, Musie Ghebremichael, Michael K. Mansour

**Affiliations:** 1 Division of Infectious Diseases, Massachusetts General Hospital, Boston, MA 02114, USA; jatallah1@bwh.harvard.edu (J.A.); dakota.archambault@maine.edu (D.A.); 2 Department of Medicine, Harvard Medical School, Boston, MA 02115, USA; kkatsis@mgh.harvard.edu (K.K.); kgallagher19@mgh.harvard.edu (K.G.); musie_ghebremichael@dfci.harvard.edu (M.G.); 3 Quantum Diamond Technologies Inc., Somerville, MA 02143, USA; jrandall@qdti.com (J.D.R.); ashepro@qdti.com (A.S.); lstyskal@qdti.com (L.E.S.); dglenn@qdti.com (D.R.G.); cconnolly@qdti.com (C.B.C.); 4 Ragon Institute of MGH, MIT and Harvard, Cambridge, MA 02138, USA

**Keywords:** IL-6, COVID-19, diagnostics, ICU, mechanical ventilation, Luminex, QDTI

## Abstract

Interleukin-6 (IL-6) has been linked to several life-threatening disease processes. Developing a point-of-care testing platform for the immediate and accurate detection of IL-6 concentrations could present a valuable tool for improving clinical management in patients with IL-6-mediated diseases. Drawing on an available biobank of samples from 35 patients hospitalized with COVID-19, a novel quantum-magnetic sensing platform is used to determine plasma IL-6 concentrations. A strong correlation was observed between IL-6 levels measured by QDTI10x and the Luminex assay (r = 0.70, *p*-value < 0.001) and between QDTI80x and Luminex (r = 0.82, *p*-value < 0.001). To validate the non-inferiority of QDTI to Luminex in terms of the accuracy of IL-6 measurement, two clinical parameters—the need for intensive care unit admission and the need for mechanical intubation—were chosen. IL-6 concentrations measured by the two assays were compared with respect to these clinical outcomes. Results demonstrated a comparative predictive performance between the two assays with a significant correlation coefficient. Conclusion: In short, the QDTI assay holds promise for implementation as a potential tool for rapid clinical decision in patients with IL-6-mediated diseases. It could also reduce healthcare costs and enable the development of future various biomolecule point-of-care tests for different clinical scenarios.

## 1. Introduction

Rapid diagnostic modalities aimed at detecting host-derived biomarkers are better able to inform clinicians on disease management [1]. A variety of technologies have evolved to meet this demand, although cost, speed, accuracy, and the ability to identify single or multiple biomarkers remain as barriers. The precise and rapid measurement of these disease-specific biomarkers can help to improve diagnostic accuracy by providing additional insight into the patient-specific immune response [2]. This information provides physicians with a greater understanding of disease pathogenesis tailored specifically to the patient, allowing for a precise and personalized treatment approach [3]. Focused medication prescription, avoidance of unnecessary testing, and better capacity for prognostication are some of the benefits of more rapid diagnosis [4]. Such improved measures ultimately lead to reduced healthcare costs and more favorable patient outcomes when confronting a variety of clinical conditions [5,6].

Biomarkers are biological molecules found in the body, and their expression levels can potentially differentiate and identify normal body conditions from abnormal life-threatening conditions [7]. Many signature biomarkers exist and have been studied in an attempt to understand their individual distinguishing properties with respect to clinical outcomes [8,9].

Interleukin-6 (IL-6), an endogenous cytokine known to elicit pro-inflammatory responses, has been linked to several disease processes [10]. IL-6-driven diseases include cytokine-release syndrome following chimeric antigen receptor (CAR) T-cell therapy [11], giant cell arteritis [12], and recently SARS-CoV-2 infection (COVID-19), mainly in severe cases associated with adverse clinical outcomes [13]. This correlation was described after the COVID-19 pandemic started in 2019. IL-6 plasma levels have been observed to serve as significant prognostic clinical indicators of COVID-19 infection. Several studies have shown that high IL-6 levels correlate with poor outcomes such as respiratory failure, high mortality rates, and the need for mechanical ventilation [14]. These findings demonstrate that IL-6 can serve as an adequate predictor for severe disease in patients infected with SARS-CoV-2, which causes COVID-19 [15,16,17]. In addition, IL-6 was proven to be a pathologic driver of inflammation related to COVID-19 infection, which is correlated with higher levels of viremia, prolonged viral RNA shedding, increased intubation, and mortality rates [18].

This correlation presented the rationale for using IL-6-blockade therapy to improve survival for COVID-19 patients in the intensive care unit (ICU) [19]. Multiple randomized controlled trials have examined the impact of IL-6 receptor blockade on the progression of SARS-CoV-2 infection. The blockade of IL-6 receptors has shown benefits in terms of survival in the ICU, but the question of utility in modifying disease outcomes if used earlier remains unknown [20,21,22,23].

The BACC Bay trial (NCT #04356937) used tocilizumab versus placebo early in COVID-19 infection to determine if IL-6 receptor blockade, prior to the need for ICU-level care and escalating supplemental oxygen, prevented disease progression. The data demonstrated that the early use of IL-6 blockade did not have a beneficial outcome [24]. We sought to compare the diagnostic performance of Luminex used to measure plasma IL-6 levels in samples from the BACC Bay trial to a novel platform for analyte measurement—the Quantum Diamond Technologies Inc (QDTI) system.

The QDTI platform utilizes quantum magnetic sensors to enable simple and rapid biomarker detection. Such biomarkers include proteins, cells, and nucleic acids using the same platform. In this study, we investigate the use of the novel QDTI platform for measuring plasma IL-6 using an existing biobank of COVID-19 plasma samples and compare its diagnostic accuracy to a traditional Luminex assay. We find that the QDTI assay demonstrates precise results when compared to the Luminex assay at a rate that can allow us to declare non-inferiority in terms of the accuracy of IL-6 measurements.

## 2. Materials and Methods

### 2.1. Patients

Our enrolled study population was distributed among five Boston hospitals. The subjects were distributed as follows: 13 patients (37%) at Massachusetts General Hospital, 9 patients (26%) at North Shore Medical Center, 6 patients (17%) at Boston Medical Center, 5 patients (14%) at Brigham and Women’s Hospital, and 2 patients (6%) at Newton-Wellesley Hospital (Table 1).

The study was conducted according to the guidelines of the Declaration of Helsinki, and approval was obtained from the MGH institutional review boards (IRB) (Approval number: 2020P001159). Informed consent was obtained from all subjects involved in the study. We collected samples from control patients (N = 35) enrolled in the double-blinded randomized controlled trial—the BACC Bay trial (NCT #04356937). These control patients have SARS-CoV-2 infection but did not receive tocilizumab treatment. The sample size was not determined statistically prior to experimentation. The blood samples of all subjects were collected on hospital admission. Serum and plasma were separated, aliquoted, and stored at −80 °C in cryovials ready for IL-6 determination. The time from venipuncture to storage was approximately 2–4 h.

### 2.2. Outcomes

The aim of this study is to compare the diagnostic performance of Luminex used to measure plasma IL-6 levels to a novel platform for analyte measurement—the Quantum Diamond Technologies Inc (QDTI) system. The primary outcomes of this study are to evaluate the correlation between IL-6 concentrations measured by both platforms and to compare the diagnostic performance of the two assays for patients who were admitted to the ICU during their hospital stay, and patients who received mechanical ventilation during their hospital stay, both of which were assumed to be correlated to worsened patient clinical outcomes [25].

### 2.3. Statistical Analysis

Statistical graphs and descriptive measures such as frequency, percent, interquartile range (IQR), and median were used to summarize data. Spearman rank correlations were used to examine bivariate associations between QDTI and Luminex assay. The Lehmann family of Receiver Operating Characteristic (ROC) curves were used to assess the diagnostic performance of QDTI and Luminex assays for worsened patient outcomes, i.e., ICU admission and use of mechanical ventilation [26]. The area under the ROC curve was used to evaluate and compare the overall diagnostic accuracy of the three assays. Bootstrap-based confidence intervals were calculated for the area under the ROC curves. All *p*-values are two-sided, and a *p* value of less than 0.05 was considered significant. Statistical analyses were performed using the R package version 4.0.3 and SAS software version 9.4 (SAS Institute, Cary, NC, USA).

### 2.4. Magnetic Imaging Assay

A quantum diamond microscope provides the core functionality of the QDTI instrument [27,28]. The system employs a synthetic diamond chip doped with quantum defects called nitrogen vacancy (NV) centers [29]. Magnetically labeled samples are placed on top of the chip and measured by detecting their magnetic fields using a thin layer of NV centers in the diamond surface. When illuminated with green light and driven with microwaves, the NV centers emit red fluorescence at a rate that depends on the local magnetic field. A camera images this fluorescence over a millimeter-scale field of view, allowing millions of independent magnetic measurements to be made simultaneously.

**Figure 1:**
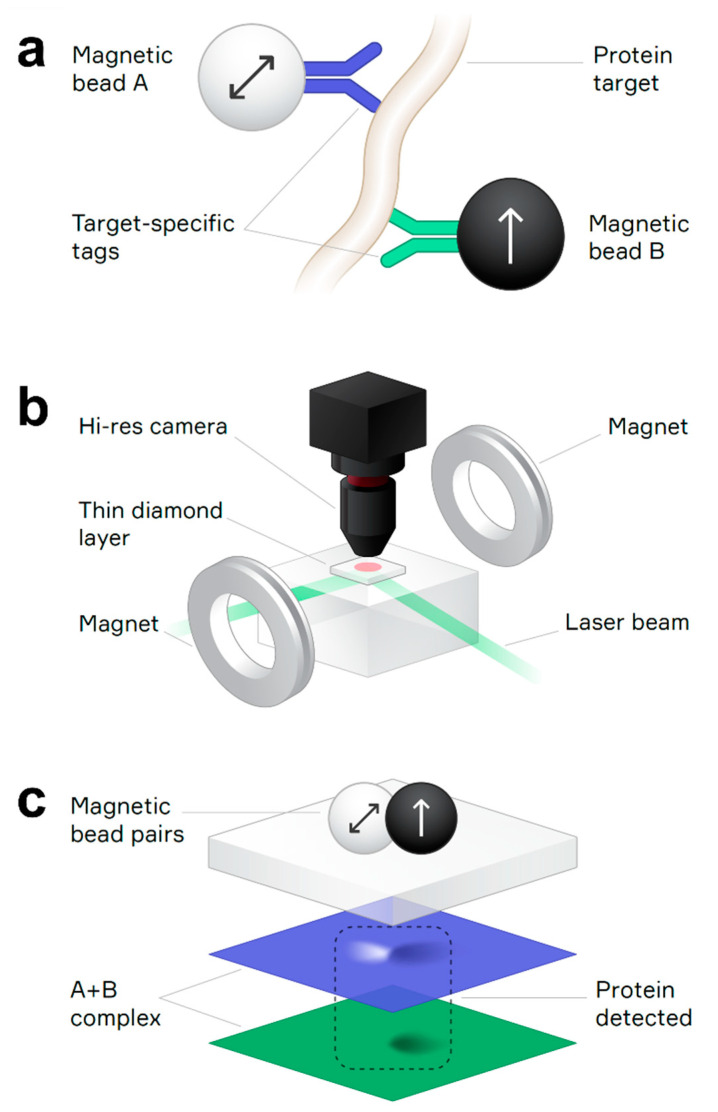
(**a**) Immunocomplex containing two magnetic beads covalently bonded to antibody tags specific to different epitopes of the target protein analyte. Arrows indicate the different magnetic properties of magnetic beads A and B, which are superparamagnetic and ferromagnetic, respectively. (**b**) Schematic of the quantum diamond microscope featuring a synthetic diamond chip with a thin layer of NV centers, a laser beam to excite NV center fluorescence, a high-resolution objective lens to collect the fluorescence onto a camera, and an electromagnet to apply different bias magnetic fields to the magnetic beads in the sample. (**c**) An immunocomplex containing both magnetic bead A and bead B is identified by overlapping magnetic dipole signals (light + dark lobes) in the separated bead A and bead B detection channels (blue and green images, respectively).

The QDTI magnetic assay counts immunocomplexes formed from pairs of magnetically distinct beads, with each bead bound to a specific epitope on the target analyte molecule (Figure 1a). The two bead types—bead A and bead B—are distinguished by their different response to applied magnetic fields. An electromagnet generates a field that can be varied between the acquisition of successive magnetic images (Figure 1b). Linear combinations of these images provide orthogonal detection channels for bead A and bead B. Immunocomplexes containing analyte molecules are identified as spatially overlapped signals in each channel (Figure 1c). Unbound beads appear as non-coincident detections and are discounted, enabling ultra-sensitive detection without the need to remove unbound bead reagents with extensive wash steps. The assay signal is the fraction of beads contained in analyte-bound immunocomplexes.

The QDTI instrument is a benchtop device for the magnetic imaging readout of samples supported in a 96-well cartridge format. The instrument processes cartridge wells sequentially, positioning each sample between the diamond chip and a microscope objective that collects NV center fluorescence. To maximize imaging resolution, samples are supported on thin polymer membranes that lie flat against the diamond surface during imaging. The membranes also provide a barrier between the chip and the samples to prevent inter-sample contamination. The acquisition time for one image is approximately one minute, including stage and pneumatic motions to position the cartridge and deflect the membrane against the diamond chip (Figure 1).

### 2.5. QDTI Assay Protocol

The assay was performed in the Division of Infectious Diseases at Massachusetts General Hospital in Boston, MA. Beads were prepared according to the manufacturer’s instructions (QDTI, Somerville, MA, USA). Calibrator samples and clinical plasma samples were plated in quadruplicate on a 96-well plate and incubated for 30 min with shaking with capture beads (bead A). The 96-well plate was placed on a bar magnet, and all liquid was aspirated before addition of detector beads (bead B). Wells were washed using wash buffer provided by QDTI and incubated on a plate shaker and bar magnet. Wash buffer was aspirated from each well, and a second wash step occurred using imaging buffer. Fresh imaging buffer was placed into each well and transferred to the QDTI provided cartridge. The total sample manipulation time after incubation required less than 30 min. The cartridge was then transferred to a dry box with desiccant for 1 h and UV-inactivated prior to loading into the instrument. Cartridges were transported in secure biosafety containers to QDTI for readout.

### 2.6. QDTI IL-6 Assay Specifications:

#### 2.6.1. Assay Sensitivity

**Figure 2:**
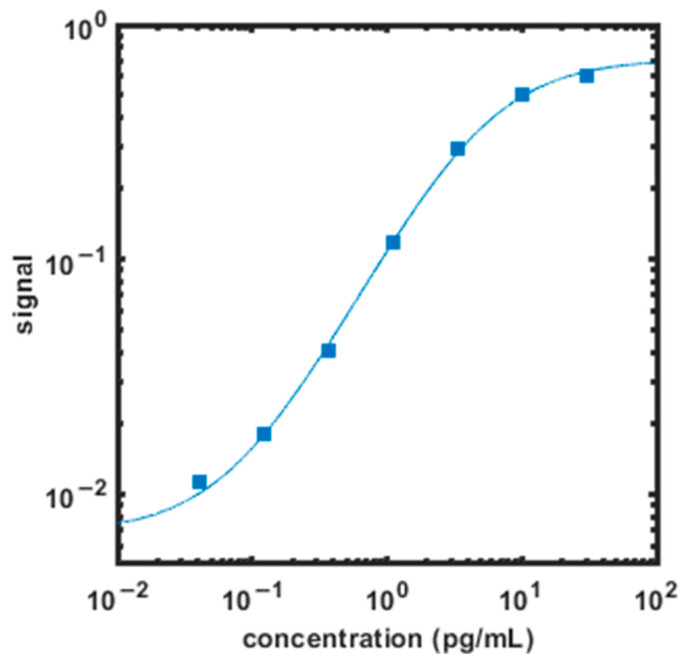
Example IL-6 assay calibration curve (squares). Assay signal is the fraction of beads contained in analyte-bound immunocomplexes. The solid line is a four-parameter logistic fit with 1/y^2^ weighting.

Calibration curves used to calculate analyte concentrations are established using a four-parameter logistic fit with 1/y^2^ weighting. The lower limit of detection (LLOD) was calculated as three standard deviations from the mean background signal (zero standard) read back on each calibration curve, averaged over seven runs. The lower limit of quantification (LLOQ) is the lowest concentration at which the median CV of calculated concentration is <25%. (Figure 2 and Table 2).

#### 2.6.2. Recovery and Linearity

To evaluate spike recovery, plasma samples were spiked with calibrator, and the increase in measured concentration was compared to the spiked concentration. Dilutional linearity was tested by spiking the calibrator into plasma samples and diluted with sample diluent. Both spike recovery and dilution linearity data are given as the mean of four experiments per sample (Table 3). Recovery and linearity testing were performed using commercial plasma samples (BioIVT, Westbury, NY, USA). The following individuals (N = 20) were neither part of the BACC Bay Trial nor part of the analysis in this study.

#### 2.6.3. Luminex

The 27-plex Luminex was performed as per the manufacturer’s instructions (Bio-techne, Minneapolis, MN, USA) with a minor deviation immediately prior to acquisition. In brief, serum samples were thawed and centrifuged at 16,000× *g* for 4 min. A seven-point standard curve was used to quantify the concentration of IL-6 in each sample. A 1 in 2 dilution of serum was performed; however, in cases where the concentration exceeded the top standard, additional dilutions were performed. At the time of testing, the potential infectious nature of serum from SARS-CoV-2 infectious patients was unknown; therefore, beads were fixed in 4% paraformaldehyde before being washed and resuspended in wash buffer and acquired on a FLEXMAP 3D.

## 3. Results

### 3.1. Patients

Plasma samples from 35 patients from the BACC Bay Trial were tested. The baseline characteristics of the patients are shown in Table 1. Nineteen patients (54%) were male. The median age was 58 (interquartile range, 45–69). The youngest patient was 26 years of age and the oldest was 82 years of age. In the overall subjects, 16 patients (46%) were Hispanic or Latino, and 19 patients (54%) were non-Hispanic. The median body-mass index of the patients at baseline (the weight in kilograms divided by the square of the height in meters) was 30.2 with an interquartile range of 25.9–33.8. In total, 40% of the patients had known diabetes mellitus, and 11% had a history of previous lung disease (e.g., asthma, COPD). A total of 28 patients (80%) had a national early warning score (NEWS) scale score ≥ 7, 2 patients (6%) had a NEWS scale score of 5–6, and 5 patients (14%) had a NEWS scale score of 1–4 [30].

A total of 24 patients (68%) were receiving supplemental oxygen at ≤6 L per minute, delivered by nasal cannula, to maintain an oxygen saturation > 92%; 2 patients (6%) were receiving supplemental ventilation at >6 L per minute, delivered by nasal cannula; 1 patient (3%) was receiving high-flow oxygen; and 7 patients (20%) were not receiving supplemental oxygen at baseline. One patient (3%) required invasive mechanical ventilation. The median concentration of ferritin was 686 ng/mL (interquartile range: 378–1346), D-dimer, 670 ng/mL (interquartile range: 399–1739), erythrocyte sedimentation rate (ESR), 68 mm/h. (interquartile range: 43–87), and white blood cell (WBC) count, 5.6 × 10^9^/L (interquartile range: 4.14–7.6 × 10^9^)

### 3.2. Comparison between QDTI Assay and Luminex Assay IL-6 Results

**Figure 3:**
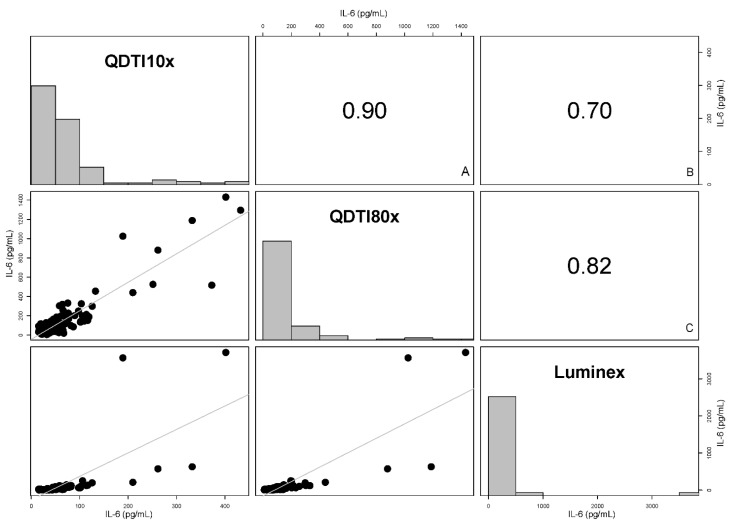
Scatter plot matrix displaying associations between IL-6 concentrations between QDTI instrument and Luminex assay. High correlations were observed between the IL-6 concentrations (**A**) between QDTI10x and QDTI80x (r = 0.90, *p*-value < 0.001) and (**B**) between the Luminex and QDTI10x (r = 0.70, *p*-value < 0.001) and (**C**) between the Luminex and QDTI80x (r = 0.82, *p*-value < 0.001).

Plasma IL-6 concentrations from all subjects were evaluated using the QDTI instrument and the Luminex assay. IL-6 concentrations were diluted 10-fold (QDTI10x), and further diluted 8-fold (QDTI80x) for the accurate measurement of IL-6 in samples that read above the calibration range at 10-fold dilution. All replicate IL-6 concentrations using single-well measurements were then determined (Supplemental Spreadsheet S1). The QDTI10x test was strongly correlated with values determined by Luminex (r = 0.70; *p*-value < 0.001) (Figure 3). Likewise, IL-6 concentrations determined using the QDTI80x test were strongly correlated with those determined by Luminex (r = 0.82; *p*-value < 0.001). As expected, strong correlation coefficient analyses were found between QDTI10x and QDTI80x. (r = 0.9; *p*-value < 0.001) (Figure 3) (Supplementary Spreadsheet S1).

**Figure 4:**
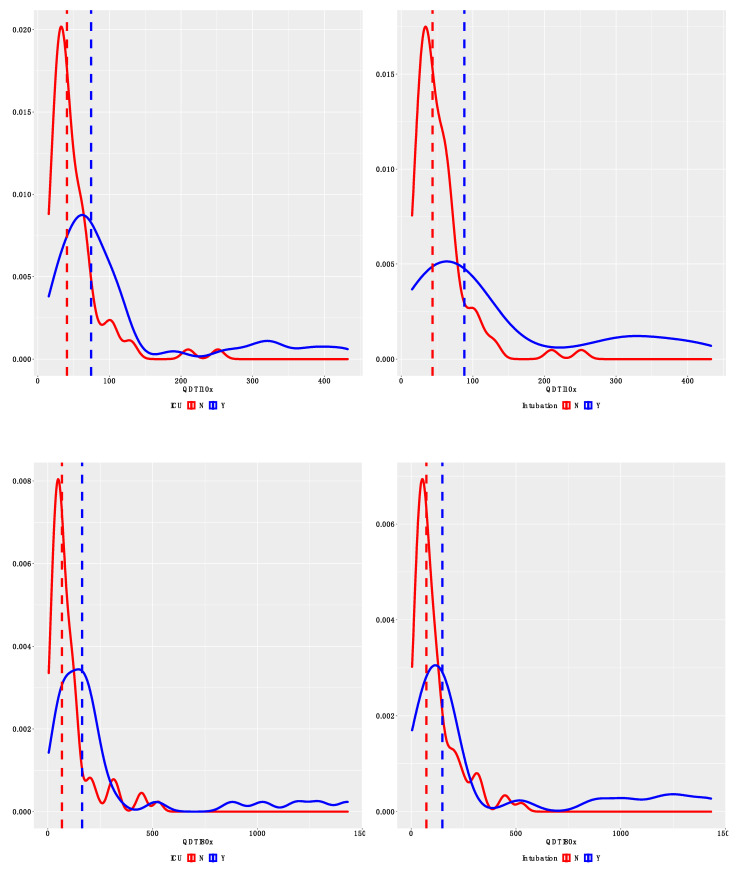

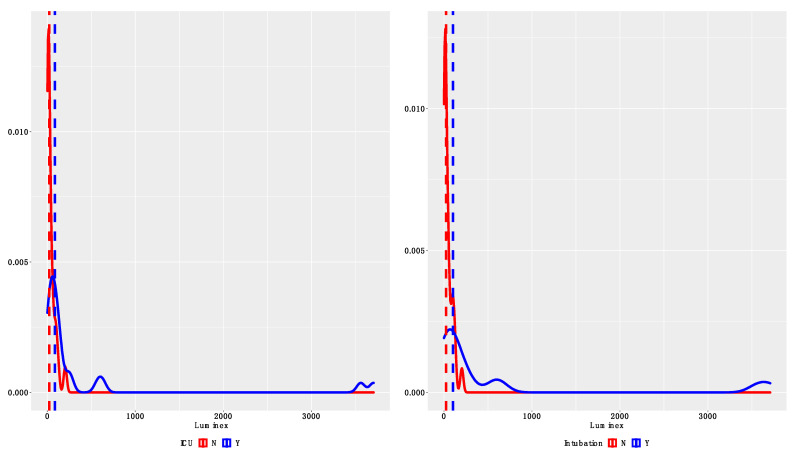
Distribution of QDTI10x, QDTI80x, and Luminex assay by ICU and intubation status. The dashed vertical lines represent median assay values. Y-axis represents density.

**Figure 5:**
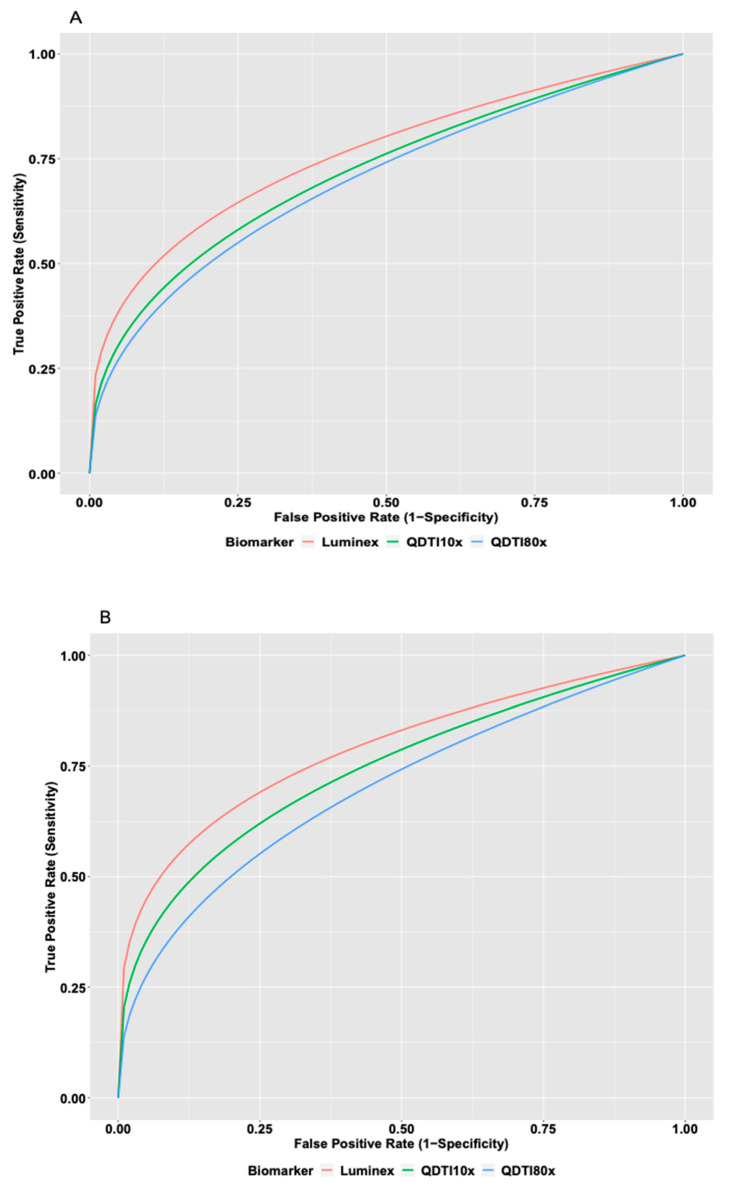
Luminex (red), QDTI10x (green), and QDTI80x (blue). (**A**) ROC for patients admitted to the ICU. (**B**) ROC for patients requiring intubation.

Receiver operating characteristic (ROC) curves were used to assess the diagnostic performance of QDTI and Luminex assays for worsened patient outcomes. Two clinical parameters, specifically ICU admission and use of mechanical ventilation, were used as measures of poor clinical outcomes. Twelve patients (34%) were admitted to the ICU during their hospital stay (Table 4). Likewise, eight patients (23%) required invasive mechanical ventilation during their hospitalization (Table 4 and Table 5). Patients requiring mechanical ventilation were also admitted to the ICU, so these patients were used in the two ROC curve analyses. For each patient, we had 2–4 replicates, and hence we had 16–48 data points for the ROC curve analyses. Figure 4 displays the distribution of QDTI10x, QDTI80x, and Luminex assay by ICU and intubation status. As can be seen from Figure 4, patients admitted to the ICU and patients requiring mechanical ventilation had higher assay values. Figure 5 displays the ROC curves for QDTI10x, QDTI80x, and Luminex assay. Results from Figure 5 show comparable receiving operating characteristic curves (ROCs) for QDTI10x, QDTI80x, and the Luminex assay. For the Luminex assay, the area under the receiver operating curve was 0.76 (95% CI: 0.65–0.86), indicating that the Luminex assay correctly identified an ICU patient from a non-ICU patient 76% of the time. Similarly, QDTI10x and QDTI80x had a 72% (AUC  =  0.72; 95% CI: 0.63–0.80) and a 70% (AUC  =  0.70; 95% CI: 0.62–0.78) probability of correctly distinguishing an ICU patient from non-ICU patient, respectively. Although the overall diagnostic accuracy of the Luminex assay was higher, there were no statistically significant differences between the ROC curves of the three assays (Figure 5A). The results for mechanical ventilation were similar, as shown in Figure 5B. More specifically, the areas under the ROC curve were 0.79 (95% CI: 0.64–0.90), 0.74 (95% CI: 0.64–0.83), and 0.70 (95% CI: 0.58–0.80) for Luminex, QDTI10x, and QDTI80x, respectively. As was the case with ICU admission, the overall diagnostic accuracy of the Luminex assay was higher, but there were no statistically significant differences between the performances of the three assays.

## 4. Discussion

In this study, a strong correlation coefficient regarding diagnostic performance has been demonstrated between the QDTI assay and the Luminex assay, indicating that the novel QDTI platform provides firm evidence of statistical equivalence as compared to Luminex. It is noteworthy to mention that IL-6 levels measured using plasma are known to be higher than those using serum [31]. The significant findings in this study provide promise for using the QDTI assay for accurate diagnostic and prognostic outcomes. The QDTI platform has the potential to serve as a point-of-care test for the accurate detection of IL-6 concentrations for patients with COVID-19 as well as other IL-6 mediated diseases [32]. Moreover, the QDTI assay had a significantly shorter turnaround time and reduced sample preparation effort. For the Luminex assay, the total sample preparation time for a 96 well plate takes approximately 6–7 h, and approximately 4.5 h for a small number of samples. Thus, the significantly quicker turnaround time of IL-6 detection by QDTI and simpler sample preparation protocol places it at an additional advantage to serve as a potential tool for rapid clinical decision making and management for patients with IL-6-mediated diseases. The QDTI assay sample preparation protocol could be further shortened considerably in an automated format targeted to few samples at the point of care.

Such rapid, high-sensitivity measurements might have the potential to result in the avoidance of unnecessary hospital stays and their associated costs and impacts for patients. Based on a recent study assessing inpatient hospital costs for COVID-19 patients in the United States, the overall median cost of a hospital stay per day is $1772, while the overall median cost of an ICU admission per day reaches $2902 [33]. The utilization of biomarkers such as IL-6 with direct implications on management could therefore potentially lead to a significant reduction in patients’ length of stay and healthcare expenditure. Additionally, the simplicity of QDTI sample preparation and implementation reduces the exhaustion of healthcare laboratory efforts [34]. Naturally, further validation of these properties is required before adopting these novel modalities for the advancement of the field of diagnostics.

Additionally, from an economic perspective, we also find that the QDTI assay has a major advantage for application in terms of lower cost. One other advantage for the QDTI assay over the Luminex assay is that the cost of Luminex is only manageable if performed in large batches, implying that it is not feasible for the generation of clinically actionable results when compared to QDTI. The complexity of Luminex execution also requires more expertise to run the assay as compared to QDTI.

It is also noteworthy that quantum magnetic sensors can be configured for the detection of additional biomolecules. The clinical scenarios in which rapid, accurate point-of-care detection of specific biomolecules could result in improved patient management are numerous. For example, developing an assay that rapidly detects and quantifies procalcitonin, a biomarker of infectious diseases, in the emergency department (ED) setting can aid in the identification and management of patients with invasive bacterial infections versus viral lower respiratory tract infections [35,36]. Another example is the management of acute stroke—the second highest leading cause of death. The management of stroke classically relies on clinical assessment and neurological imaging methods, imposing a need for more time-sensitive techniques such as point-of-care tests capable of detecting specific stroke biomarkers such as B-type Natriuretic Peptide (BNP) for suspected ischemic stroke, neuron specific enolase (NSE), fibronectin for hemorrhagic stroke, and purines (e.g., adenosine, inosine and hypoxanthine) released after stroke onset [37,38,39,40]. Several novel assays are being developed and can potentially facilitate rapid acute stroke diagnosis assisting in efficient stroke triage and treatment [41]. Moreover, point-of-care testing may prove useful in managing bleeding complications following cardiac surgery. Using point-of-care tests that detect prothrombin time, activated partial thromboplastin time, fibrinogen, and platelet function can aid in clinical decision-making on coagulation interventions for bleeding complications more rapidly than standard laboratory tests [42].

Finally, the simplicity of execution and rapid turnaround time of modalities such as quantum sensing would facilitate their adoption as point-of-care tests in healthcare settings with limited or no access to clinical laboratories and other resources [43,44,45].

In short, the early diagnosis of disease remains crucial for optimal patient management and outcomes as well as reducing healthcare costs. Rapid diagnosis ultimately results in precise clinical decisions, directed treatment plans, and improved prognosticating capacity [46]. From an economic perspective, accurate and speedy diagnostics are strongly associated with the prevention of unnecessary testing and imaging, reductions in hospital stays, and a decreased adverse events rate. Such implications provide a cost-effectiveness plan potential that would alleviate much of the overall financial burden on the healthcare industry [47,48].

## 5. Limitations

As with any study focusing on novel assays, the results presented in this manuscript have shortcomings. An important limitation in our study is the lack of an adequate sample size. Due to the limited sample size, we did not have the power to perform multivariate analyses. Multivariate analyses are required to adjust for the effect of confounding variables while comparing the three assays. Larger cohorts in prospective studies are required for the validation of the results presented in this manuscript. Moreover, the data presented in this manuscript are based on a single study center. A larger multi-center study might be adequate to achieve a larger sample size.

Another limitation to the study is the focus on one type of infection, which is SARS-CoV-2, and the absence of a diversity of IL-6-mediated diseases. Future studies and clinical trials will be required to evaluate this novel assay’s performance with respect to multiple disease states.

Finally, IL-6 concentrations for all subjects participating in this study were measured using blood collected at hospital admission. Serial measurements would be useful to evaluate the assay’s performance with respect to modifications of IL-6 levels over time and clinical outcomes.

## 6. Conclusions

In conclusion, from a technical and analytical perspective, the results of this study show that the QDTI assay proves to be non-inferior to the Luminex assay in terms of accurate IL-6 measurement for patients hospitalized with COVID-19. This novel platform also shows more rapid generation of IL-6 concentrations when compared to the Luminex assay. Future validation studies would ultimately be needed before presenting any clinical implications of the presented findings.

## Figures and Tables

**Table 1:** Patient demographics and characteristics.

Characteristic	Patients (N = 35)
Age in years (Median, IQR)	58 (45–69)
Age > 65—no. (%)	10 (28.6)
Sex—no. (%) Male Female	19 (54) 16 (46)
* Ethnic group—no. (‡%) Hispanic Non-Hispanic	16 (46) 19 (54)
BMI (Median, IQR)	30.2 (25.9–33.8)
† BMI > 30—no. (%)	18 (51)
Diabetes—no. (%)	14 (40)
History of lung disease (COPD, Asthma)—no. (%)	4 (11)
NEWS scale score—no. (%) 1–4 5–6 >7	5 (14) 2 (6) 28 (80)
Oxygen requirement at day 1—no. (%) No oxygen required Nasal Cannula 2–6 L/min Nasal Cannula > 6 L/min High Flow Nasal Cannula or non-invasive ventilation Intubation	7 (20) 24 (68) 2 (6) 1 (3) 1 (3)
Median Laboratory Values (IQR) Erythrocyte sedimentation rate—mm/hr. D-Dimer Level—ng/mL Ferritin level—ng/mL WBC count—×10^9^/L	68 (43–87) 670 (399–1739) 686 (378–1346) 5.6 (4.14–7.6)

* Ethnic groups were reported by the patients. † The body-mass index (BMI) is the weight in kilograms divided by the square of the height in meters. ‡ Percentages may not total 100 because of rounding. IQR denotes interquartile range.

**Table 2:** Human IL-6 assay sensitivity.

Human IL-6 Assay	Concentration
LLOD	0.06 pg/mL
LLOQ	0.12 pg/mL
Sample Volume	5 uLs

**Table 3:** Recovery and linearity.

	Dilution	Median	Interquartile Range
Spike Recovery (N = 4)	10×	96%	92–99%
Dilutional Linearity (N = 16)	5–10×	107%	99–119%

**Table 4:** IL-6 mean concentrations by Luminex, QDTI10x, and QDTI80x for patients admitted to the ICU.

	Luminex		QDTI 10× Dilution		QDTI 80× Dilution	
Sample Number	Median (pg/mL)	Interquartile Range (IQR)	Median (pg/mL)	Interquartile Range (IQR)	Median (pg/mL)	Interquartile Range (IQR)
106	136.25	13.50	103.05	36.61	151.82	
212	249.40	5.10	105.96		196.84	
252	3634.97	146.30	346.96	132.29	1025.27	
350	123	1.12	89.89	26.55	230.39	
355	45.10		49.075	10.46	163.83	
433	10.20	0.42	25.19		31.34	6.98
443	73.40	3.12	58.63	15.02	122.49	
601	27	0.80	61.15		76.98	29.67
608	10	0.19	24.67	3.52	77	
628	111	1.91	81.97	32.26	133.91	
679	602	55.48	314.94	102.91	1187.66	
737	98	4.87	65.65		241.68	86.05

**Table 5:** IL-6 mean concentrations by Luminex, QDTI10x, and QDTI80x for patients requiring mechanical ventilation.

	Luminex		QDTI 10× Dilution		QDTI 80× Dilution	
Sample Number	Median (pg/mL)	Interquartile Range (IQR)	Median (pg/mL)	Interquartile Range (IQR)	Median (pg/mL)	Interquartile Range (IQR)
106	136.25	13.50	103.05	36.61	151.82	
212	249.40	5.10	105.96		196.84	
252	3634.97	146.30	346.96	132.29	1025.27	
433	10.20	0.42	25.19		31.34	6.98
443	73.4.	3.12	58.63	15.02	122.49	
608	10	0.19	24.67	3.52	77.0	
628	111	1.91	81.97	32.36	133.91	
679	602	55.48	314.94	102.91	1187.66	

## Data Availability

The data presented in this study are available in the article and Supplementary Material Spreadsheet S1.

## Supplementary Materials

The following supporting information can be downloaded at https://www.mdpi.com/article/10.3390/diagnostics12051164/s1, Spreadsheet S1: Raw values of IL-6 measured by the Luminex assay, QDTI10x, and QDTI80x.

## Abbreviations

ACS	Acute coronary syndrome
CAR-T cell therapy	Chimeric antigen receptor-T cell therapy
CRP	C-reactive protein
ED	Emergency department
ESR	Erythrocyte sedimentation rate
ICU	Intensive care unit
IL-6	Interleukin-6
IQR	Interquartile range
LLOD	Lower limit of detection
LLOQ	Lower limit of quantification
NEWS	National early warning score
NV	Nitrogen vacancy
ROC	Receiving operating characteristic curve
WBC	White blood cells
